# Supplementary material for: Determinants of lifestyle counseling and current practices: A cross-sectional study among Dutch general practitioners
Source: PLoS One. 2020 Jul 21;15(7):e0235968. doi: 10.1371/journal.pone.0235968 (PMC7373284; doi:10.1371/journal.pone.0235968)
Supplement: S1 File — (PDF) [file pone.0235968.s001.pdf]

S1 File.

Leefstijl in de huisartsenpraktijk

Welkom bij dit onderzoek en dank u voor uw deelname!

Deze vragenlijst gaat over **leefstijl in de huisartsenpraktijk** en zal ongeveer 10 minuten in beslag nemen. De **doelgroep** van deze vragenlijst **betreft huisartsen** in Nederland.

Het doel van dit onderzoek is inzicht te krijgen in **hoe huisartsen staan ten opzichte van leefstijl**. **Iedere 25e respondent** die de vragenlijst invult, ontvangt een **cadeaubon ter waarde van 25 euro**.
Uw gegevens zullen **anoniem** worden verwerkt. Indien u contact wenst op te nemen met de onderzoeker om dit onderzoek te **bespreken** of **de resultaten van deze studie wilt ontvangen**, dan kunt u een e-mail sturen naar info@artsenleefstijl.nl.

Toestemming
Door de vragenlijst te beginnen gaat u ermee akkoord dat u op **vrijwillige en anonieme basis** aan deze studie deelneemt, u weet dat u zich kunt **terugtrekken** uit deze studie en dat uw antwoorden worden gebruikt in **wetenschappelijk onderzoek.**

- ○ Ja ik ga hiermee akkoord
- ○ Nee ik ga hiermee niet akkoord

In deze vragenlijst wordt er onder **leefstijl** het volgende verstaan: 'Leefstijl is een verzamelnaam voor het gedrag van een persoon met betrekking tot voeding, beweging, slaap, stress, roken en alcoholgebruik.'

Leefstijl bespreken

Onder bespreken wordt het volgende verstaan: 'Tijdens een consult actief aandacht geven aan leefstijl, waarbij met de patiënt inzicht wordt verkregen in diens leefstijlgedrag.'

Q2 'Ik vind het bespreken van leefstijl...' Geef de plek aan die op u van toepassing is

Leuk	○	○	○	○	○	○	○	Vervelend
Makkelijk	○	○	○	○	○	○	○	Moeilijk
Motiverend	○	○	○	○	○	○	○	Demotiverend
Niet ongemakkelijk/niet gênant	○	○	○	○	○	○	○	Ongemakkelijk/gênant

Q3 'Ik vind het bespreken van leefstijl... ' Geef de plek aan die op u van toepassing is

Een van mijn taken als huisarts	○	○	○	○	○	○	○	Niet een van mijn taken als huisarts
Nuttig	○	○	○	○	○	○	○	Nutteloos
Belangrijk om de gezondheid van mijn patiënten te verbeteren	○	○	○	○	○	○	○	Onbelangrijk om de gezondheid van mijn patiënten te verbeteren

Q4 Hoe vaak vraagt u patiënten...

	Nooit	Zelden	Soms	Vaak	Altijd
Naar hun leefstijl	○	○	○	○	○
Of ze gemotiveerd	○	○	○	○	○

zijn om hun leefstijl te verbeteren?

Q5 Hoe makkelijk vindt u het om de volgende leefstijlgewoonten te bespreken?

	Heel erg makkelijk	Makkelijk	Niet makkelijk/Niet moeilijk	Moeilijk	Heel erg moeilijk
Roken	○	○	○	○	○
Alcohol gebruik	○	○	○	○	○
Voeding	○	○	○	○	○
Beweging	○	○	○	○	○
Slapen	○	○	○	○	○
Stress	○	○	○	○	○

Q6 Geef uw mening over elk van de onderstaande stellingen

	Helemaal mee oneens	Oneens	Noch oneens/ noch eens	Eens	Helemaal mee eens
Huisartsen horen leefstijl te bespreken	○	○	○	○	○
Ik denk dat andere huisartsen leefstijl bespreken	○	○	○	○	○
Ik denk dat patiënten van mij verwachten dat ik leefstijl bespreek	○	○	○	○	○

Q7 Hoe vaak bespreekt u de volgende factoren die voor patiënten (mogelijk) een **belemmering** vormen voor een gezonde leefstijl?

	Nooit	Zelden	Soms	Vaak	Altijd

Stress	○	○	○	○	○
Verleidingen	○	○	○	○	○
Gebrek aan tijd	○	○	○	○	○
Gebrek aan kennis	○	○	○	○	○
Gebrek aan motivatie	○	○	○	○	○
Gebrek aan financiële middelen	○	○	○	○	○
Gebrek aan (zelf) vertrouwen	○	○	○	○	○

Anders, namelijk

Q8 In welke mate kent u de richtlijn dagelijkse hoeveelheid groenten en fruit van het **Voedingscentrum**?

○ Helemaal niet

○ Nauwelijks

○ In redelijke mate

○ In hoge mate

○ In zeer hoge mate

Q9 Neemt u voedingsanamneses af bij uw patiënten?

○ Ja

○ Nee

Deze vraag weergeven:
If Q9 = Ja

Q10 In geval van welke aandoeningen neemt u een voedingsanamnese af? (U kunt meerdere opties aankruisen)

☐ Hypertensie

- ☐ Hypercholesterolemie
- ☐ Obesitas/overgewicht
- ☐ Diabetes
- ☐ Darmklachten
- ☐ Kanker
- ☐ Hart-en vaatziekten
- ☐ Anders, namelijk____

Q11 Bespreekt u de groente-en fruitconsumptie van uw patiënten?

- ○ Nee
- ○ Ja ik vraag er naar als ik denk dat dat relevant is
- ○ Ja, als uit de voedingsanamnese blijkt dat een patiënt te weinig groenten en fruit eet

Adviseren

Onder **adviseren** wordt het volgende verstaan: 'Het geven van persoonlijke raad aan patiënten over hun leefstijl, inclusief het geven van informatie over de gezondheidsrisico's en gezondheidsvoordelen om gedragsverandering te stimuleren.'

Q12 Hoe **vaak** geeft u op basis van een bespreking van de leefstijl, advies aan uw patiënten bij de volgende leefstijlgewoonten?

	Nooit	Zelden	Soms	Vaak	Altijd
Roken	○	○	○	○	○
Alcohol gebruik	○	○	○	○	○
Voeding	○	○	○	○	○
Beweging	○	○	○	○	○
Slapen	○	○	○	○	○
Stress	○	○	○	○	○

Q13 Geef uw mening over elk van de onderstaande stellingen

	Niet(s)	Nauwelijks	In redelijke mate	In hoge mate	In zeer hoge mate
Ik kan patiënten motiveren hun leefstijl te verbeteren	○	○	○	○	○
Ik kan patiënten handvatten bieden om hun leefstijl te verbeteren	○	○	○	○	○

Q14 Wanneer u uw patiënten adviezen geeft, hoe vaak stelt u dan samen met hen **concrete doelen** op om de volgende leefstijlgewoonten te veranderen?

	Nooit	Zelden	Soms	Vaak	Altijd
Roken	○	○	○	○	○
Alcohol gebruik	○	○	○	○	○
Voeding	○	○	○	○	○
Beweging	○	○	○	○	○
Slapen	○	○	○	○	○
Stress	○	○	○	○	○

Q15 Hoe vaak geeft u uw patiënten adviezen over het eten van groenten en fruit?

○ Helemaal nooit

○ Nauwelijks

○ Soms

○ Vaak

○ Heel vaak

Q16 Welke **dagelijkse** hoeveelheid groenten en fruit adviseert u aan uw patiënten? (groenten in grammen, fruit in stuks)

○ Groenten ____

○ Fruit ____

Q17 Wat doet u als een patiënt een **te lage inname** heeft van groenten en fruit? **''Ik...''** (U kunt meerdere opties aankruisen)

- ☐ Vraag aan de patiënt naar de oorzaak hiervan
- ☐ Noem de aanbevolen hoeveelheid groenten en fruit
- ☐ Geef advies over hoe de groenten-en fruitconsumptie verhoogd kan worden
- ☐ Verschaffen van informatiemateriaal (bijv. een folder)
- ☐ Doorverwijzen naar een dietist
- ☐ Doorverwijzen naar een verpleegkundige / POH’er

Anders, namelijk____

Barrières, wensen en behoeften

Q18 'Ik zou leefstijl vaker willen bespreken/adviseren dan ik nu doe'

- ○ Ja
- ○ Nee

Deze vraag weergeven:
If Q18 = Ja

Q19 "Ik bespreek/adviseer leefstijl **minder vaak dan ik zou willen** door..."

	Helemaal mee oneens				Helemaal mee eens
Mijn gebrek aan motivatie	○	○	○	○	○
Gebrek aan tijd	○	○	○	○	○
Gebrek aan kennis	○	○	○	○	○
Gebrek aan zelfvertrouwen	○	○	○	○	○
Gebrek aan financiële vergoeding	○	○	○	○	○
Gebrek aan bewijs / richtlijn	○	○	○	○	○
Gebrek aan middelen ter ondersteuning	○	○	○	○	○

Gebrek aan overzicht van verwijsmogelijkheden	○	○	○	○	○
Patiënten die hier niet op zitten te wachten	○	○	○	○	○
Het lage opleidingsniveau van sommige patiënten	○	○	○	○	○
Angst om de relatie met de patiënt te verstoren	○	○	○	○	○
Ongemotiveerde patiënten	○	○	○	○	○
Taal/cultuurbarrière bij mijn patiënten	○	○	○	○	○
Andere problematiek die speelt bij patiënten	○	○	○	○	○
De huidige inrichting van ons gezondheidssysteem	○	○	○	○	○

Deze vraag weergeven:
If Q18 = Ja

Anders, namelijk

____

Deze vraag weergeven:
If Q18 = Ja

Q20 Wat zou u het **meeste motiveren of helpen** om vaker leefstijl te bespreken/adviseren? (Gebruik minimaal 10 tekens)

____

Q21 Waaraan zou u behoefte hebben om in uw geval groenten en fruit tijdens een consult vaker te kunnen bespreken?

	Helemaal mee oneens	Oneens	Noch oneens/ noch eens	Eens	Helemaal mee eens
Ik heb deze behoefte niet	○	○	○	○	○

Meer kennis over het belang van groenten en fruit	○	○	○	○	○
Wetenschappelijk bewijs voor de effecten van groenten en fruit	○	○	○	○	○
Succesverhalen van andere huisartsen	○	○	○	○	○
Tools ter ondersteuning	○	○	○	○	○
NHG richtlijnen over groenten en fruit	○	○	○	○	○
Samenwerking met bijvoorbeeld de lokale supermarkt/ groenteboer	○	○	○	○	○
Anders, namelijk					

Q22 Ervaart u een of meerdere factoren die een **barrière** vormen voor het goed bespreken/adviseren van groenten en fruit?

○ Ja

○ Nee

Deze vraag weergeven:
If Q22 = Ja

Q23 Welke factor(en) ervaart u die een barrière vormt(en) voor het goed bespreken/adviseren van groenten en fruit? (gebruik minimaal 10 tekens)

Q24 Welke van de onderstaande opties zijn wellicht interessant voor uw praktijk om uw patiënten te stimuleren tot het eten van groenten en fruit? (U kunt meerdere opties aanvinken)

☐ Kortingsbonnen voor groenten en fruit voor mijn patiënten bij bijvoorbeeld supermarkten/ groenteboeren

☐ Gratis snackgroenten in de wacht- of spreekkamer

- ☐ Informatie over het belang van groenten en fruit (folders, filmpjes)
- ☐ Meer standaard aanbiedingen op groenten en fruit, bijvoorbeeld in supermarkten
- ☐ Meer nudges (uitlokken van gedrag) gericht op groenten en fruit in supermarkten (kleuren, afbeeldingen, schapindeling)
- ☐ Betere communicatie (informatie over groenten en fruit, recepten, demonstraties)
- ☐ Geen van allen

Anders, namelijk

____

Doorverwijzen en nazorg

Q25 Hoe vaak verwijst u uw patiënten door naar andere professionals om hun leefstijl te verbeteren?

- ○ Nooit
- ○ Zelden
- ○ Soms
- ○ Vaak
- ○ Altijd

Q26 Wanneer u uw patiënten doorverwijst ter verbetering van hun leefstijl, hoe vaak verwijst u hen dan door naar de volgende zorgprofessionals?

	Nooit	Zelden	Soms	Vaak	Altijd
Fysiotherapeut	○	○	○	○	○
Diëtist	○	○	○	○	○
Praktijkondersteuner / praktijkverpleegkundige	○	○	○	○	○
Geregistreerd leefstijlcoach (BLCN)	○	○	○	○	○
Psycholoog	○	○	○	○	○
Medisch specialist	○	○	○	○	○

Q27 Is groente- en fruitconsumptie een onderwerp dat ter sprake komt in uw afstemming met diëtisten en POH'ers?

- ○ Ja

○ Nee

Q28 Bent u bekend met het feit dat u per 1 januari 2019 patiënten kunt doorverwijzen naar gecombineerde leefstijlinterventies (GLI's)?

○ Ja

○ Nee

Q29 Verwijst u naar GLI's?

○ Ja

○ Nee

Q30 "Als ik patiënten **niet** of **niet nog meer dan ik zou willen** naar GLI's verwijs, dan komt dit door...

	Helemaal mee oneens	Oneens	Noch oneens/ noch eens	Eens	Helemaal mee eens
Gebrek aan kennis over GLI's	○	○	○	○	○
Gebrek aan vertrouwen in GLI's	○	○	○	○	○
Gebrek aan tijd	○	○	○	○	○
Gebrek aan motivatie	○	○	○	○	○
Gebrek aan GLI's in de buurt	○	○	○	○	○
Onzekerheid over financiële vergoeding in de toekomst	○	○	○	○	○
Voorkeur voor doorverwijzing naar bekende professionals binnen of buiten mijn praktijk	○	○	○	○	○

Anders, namelijk

Q31 Wanneer u uw patiënten leefstijladviezen geeft, hoe vaak levert u dan **nazorg** aan hen, bijvoorbeeld vervolgafspraak, nabellen, medicatie afbouw?

- Nooit
- Zelden
- Soms
- Vaak
- Altijd

Q32 Wanneer uw patiënten een leefstijlinterventie ondergaan, hoe vaak bouwt u dan medicatie af?

- Nooit
- Zelden
- Soms
- Vaak
- Altijd

Q33 Welk cijfer geeft u uw eigen leefstijl?

- 1
- 2
- 3
- 4
- 5
- 6
- 7
- 8
- 9
- 10

Algemene vragen (De vragenlijst duurt nu nog minder dan een halve minuut)

Q34 Wat is uw geslacht?

- ◯ Vrouw
- ◯ Man

Q35 Wat is uw leeftijd?

____

Q36 Hoeveel dagdelen bent u gemiddeld werkzaam als huisarts? (1 dag = 2 dagdelen)

____

Q37 Wat is de postcode van uw huisartsenpraktijk? (enkel de cijfers)

____

Q38 Bent u werkzaam in een...

- ◯ Solopraktijk
- ◯ Solopraktijk in gezondheidscentrum
- ◯ Duopraktijk zelfstandig
- ◯ Duopraktijk in gezondheidscentrum
- ◯ Groepspraktijk zelfstandig
- ◯ Groepspraktijk in gezondheidscentrum

Q39 Kent u Vereniging Arts & Leefstijl? (voorheen Arts en Voeding)

- ◯ Nee ik ken het niet
- ◯ Ja ik ken het, maar geen lid en niet naar bijscholing geweest
- ◯ Ja ik ken het, wel lid, maar niet naar bijscholing geweest
- ◯ Ja ik ken het, lid én naar een bijscholing geweest

Dit is het einde van de vragenlijst. Wanneer u kans wilt maken op de cadeaubon van 25 euro, dan kunt u hieronder uw e-mailadres achterlaten.

____
