# Supplementary material for: Determinants of lifestyle counseling and current practices: A cross-sectional study among Dutch general practitioners
Source: PLoS One. 2020 Jul 21;15(7):e0235968. doi: 10.1371/journal.pone.0235968 (PMC7373284; doi:10.1371/journal.pone.0235968)
Supplement: S2 File — (PDF) [file pone.0235968.s002.pdf]

S2 File

Lifestyle in the general practice

Welcome to this study and thank you for your participation.

This questionnaire is about **lifestyle in general practice** and will take about 10 minutes to fill out.
The **target group** of this questionnaire is **general practitioners** (GPs) in the Netherlands.

The aim of this study is to gain insight into **how GPs relate to lifestyle**. **Every 25th respondent** who completes the questionnaire receives a gift voucher worth 25 euros.
Your data will be processed anonymously. If you wish to contact the researcher **to discuss this research or receive the results** of this study, you can send an email to info@artsenleefstijl.nl.

Permission
By starting the questionnaire, you agree to participate in this study on a **voluntary and anonymous basis**, knowing that you can **withdraw** from this study and that your answers will be used in **scientific research.**

◯ Yes I agree with this

◯ No I do not agree with this

In this questionnaire, **lifestyle** means the following: 'Lifestyle is a collective name for a person's behaviour with regard to nutrition, physical activity, sleep, stress, smoking and alcohol use.'

Discussing lifestyle
Discussing lifestyle means the following : "Giving active attention to lifestyle during a consultation, whereby the patient gains insight into his lifestyle behaviour."

Q2 ‘I think discussing lifestyle is...' Indicate the place that applies to you

Nice	○	○	○	○	○	○	○	Annoying
Easy	○	○	○	○	○	○	○	Difficult
Motivating	○	○	○	○	○	○	○	Demotivating
Not uncomfortable / not embarrassing	○	○	○	○	○	○	○	Uncomfortable / embarrassing

Q3 'I think discussing lifestyle is... ' Indicate the place that applies to you

One of my tasks a GP	○	○	○	○	○	○	○	Not one of my tasks as a GP
Useful	○	○	○	○	○	○	○	Useless
Important to improve the health of my patients	○	○	○	○	○	○	○	Unimportant to improve the health of my patients

Q4 How often do you ask your patients...

	Never	Rarely	Sometimes	Very often	Always
About their lifestyle?	○	○	○	○	○
Whether they are motivated to change their lifestyle?	○	○	○	○	○

Q5 How easy do you find it to discuss the following lifestyle habits?

	Very easy	Easy	Neutral	Difficult	Very difficult
Smoking	○	○	○	○	○
Alcohol use	○	○	○	○	○
Nutrition	○	○	○	○	○
Physical activity	○	○	○	○	○
Sleep	○	○	○	○	○
Stress	○	○	○	○	○

Q6 Give your opinion on each of the statements below

	Strongly disagree	Disagree	Neutral	Agree	Totally agree
GPs should discuss lifestyle	○	○	○	○	○
I believe other GPs discuss lifestyle	○	○	○	○	○
I believe patients expect me to discuss lifestyle	○	○	○	○	○

Q7 How often do you discuss the following factors that may (possibly) present a **barrier** to a healthy lifestyle for patients?

	Never	Barely	Sometimes	Often	Always
Stress	○	○	○	○	○
Temptations	○	○	○	○	○

Lack of time	○	○	○	○	○
Lack of knowledge	○	○	○	○	○
Lack of motivation	○	○	○	○	○
Lack of financial resources	○	○	○	○	○
Lack of confidence	○	○	○	○	○

Otherwise, namely
____

Q8 To what extent do you know the guideline daily amount of fruit and vegetables of the **Voedingscentrum**?

○ Not at all

○ Barely

○ To a reasonable degree

○ To a high degree

○ To a very high degree

Q9 Do you take a nutritional history at your patients?

○ Yes

○ No

Display this question:
If Q9 = Yes

Q10 In case of which disease do you take a nutritional history? (You can tick multiple options)

▢ Hypertension

▢ Hypercholesterolemia

- [ ] Obesity / overweight
- [ ] Diabetes
- [ ] Intestinal complaints
- [ ] Cancer
- [ ] Heart and vascular diseases
- [ ] Otherwise, namely____

Q11 Do you discuss the fruit and vegetable consumption of your patients?

○ No

○ Yes, I ask to it if I think it is relevant

○ Yes, if the nutritional history shows that a patient is not eating enough fruit and vegetables

Advising lifestyle

Advising lifestyle means the following: 'Giving patients personal advice on their lifestyle, including providing information on health risks and health benefits to encourage behavioural change.'

Q12 Based on the assessment, how **often** do you advise your patients on the following lifestyle habits?

	Never	Rarely	Sometimes	Often	Always
Smoking	○	○	○	○	○
Alcohol use	○	○	○	○	○
Nutrition	○	○	○	○	○
Physical activity	○	○	○	○	○
Sleep	○	○	○	○	○
Stress	○	○	○	○	○

Q13 Give your opinion on each of the statements below

	Not at all	Barely	To a reasonable degree	To a high degree	To a very high degree
I can motivate patients to improving their lifestyle	○	○	○	○	○
I can offer my patients tools to improve their lifestyle	○	○	○	○	○

Q14 When you advise your patients, how **often** do you set concrete goals together to change the following lifestyle habits?

	Never	Barely	Sometimes	Often	Always
Smoking	○	○	○	○	○
Alcohol use	○	○	○	○	○
Nutrition	○	○	○	○	○
Physical activity	○	○	○	○	○
Sleep	○	○	○	○	○
Stress	○	○	○	○	○

Q15 How **often** do you advise your patients about eating fruits and vegetables?

○ Never

○ Barely

○ Sometimes

○ Often

○ Very often

Q16 What **daily** amount of fruit and vegetables do you recommend to your patients? (vegetables in grams, fruit in pieces)

○ Vegetables ____

○ Fruit ____

Q17 What do you do if a patient has a **too low** intake of fruit and vegetables? "" **I ...** "" (You can check multiple options)

☐ Ask the patient for the cause of this

☐ Mention the recommended amount of fruits and vegetables

☐ Provide advice on how to increase fruit and vegetable consumption

☐ Provide information material (e.g. leaflet)

☐ Refer to a dietician

☐ Refer to a nurse / practice assistant

☐ Otherwise, namely____

Barriers, wishes and needs

Q18 'I would like to discuss / advise lifestyle more often than I do now'

○ Yes

○ No

Display this question:
If Q18 = Yes

Q19 'I discuss / advise lifestyle **less often** than I would like by ...'

	Totally disagree	Totally agree

My lack of motivation	○	○	○	○	○
Lack of time	○	○	○	○	○
Lack of knowledge	○	○	○	○	○
Lack of confidence	○	○	○	○	○
Lack of financial compensation	○	○	○	○	○
Lack of prove / guideline	○	○	○	○	○
Lack of supporting tools	○	○	○	○	○
Lack of overview of referring possibilities	○	○	○	○	○
Patients who do not need this	○	○	○	○	○
The low education level of some patients	○	○	○	○	○
Fair to disrupt the relation with the patient	○	○	○	○	○
Unmotivated patients	○	○	○	○	○
Language / culture barrier in my patients	○	○	○	○	○
Other problems that affect patients	○	○	○	○	○

The current structure of our health system	○	○	○	○	○

Display this question:
If Q18 = Yes

Otherwise, namely

____

Display this question::
If Q18 = Yes

Q20 What would **motivate you most or help** you to discuss / advise lifestyle more often? (Use at least 10 characters)

____

Q21 What would you need to be able to discuss fruits and vegetables **more often** during a consultation?

	Totally disagree	Disagree	Neutral	Agree	Totally agree
I do not have this need	○	○	○	○	○
More knowledge about the importance of fruit and vegetables	○	○	○	○	○
Scientific evidence for the effects of fruits and vegetables	○	○	○	○	○
Success stories of other GPs	○	○	○	○	○
Supporting tools	○	○	○	○	○
	○	○	○	○	○

NHG guidelines about fruit and vegetables					
Cooperation with, for example, the local supermarket / grocery	○	○	○	○	○

Otherwise, namely ____

Q22 Are you experiencing one or more factors that **hinder** the proper discussion / advice of fruit and vegetables?

○ Yes

○ No

Display this question:
If Q22 = Yes

Q23 What factor(s) do you experience that form(s) a **barrier** for the proper discussion / advice of fruit and vegetables? (use at least 10 characters)

____

Q24 Which of the following options might be interesting for your practice to encourage your patients to eat fruits and vegetables? (You can check multiple options)

▢ Discount coupons for fruit and vegetables for my patients, for example, at supermarkets / greengrocers

▢ Free snack vegetables in the waiting or consultation room

▢ Information about the importance of fruit and vegetables (leaflets, video's)

▢ More standard offers on fruit and vegetables, for example in supermarkets

▢ More nudges (provoking behaviour) aimed at fruit and vegetables in supermarkets (colours, images, shelf layout)

▢ Better communication (information on fruit and vegetables, recipes, demonstrations)

☐ None of them

Otherwise, namely

____

Referring and follow-up support

Q25 How **often** do you refer your patients to other professionals to improve their lifestyle?

- ◯ Never
- ◯ Barely
- ◯ Sometimes
- ◯ Often
- ◯ Always

Q26 When you refer your patients to improve their lifestyle, how **often** do you refer them to the following healthcare professionals?

	Never	Barely	Sometimes	Often	Always
Physiotherapist	◯	◯	◯	◯	◯
Dietician	◯	◯	◯	◯	◯
Practice assistant / nurse	◯	◯	◯	◯	◯
Registered lifestyle coach (BLCN)	◯	◯	◯	◯	◯
Psychologist	◯	◯	◯	◯	◯
Medical doctor	◯	◯	◯	◯	◯

Q27 Is fruit and vegetable consumption a subject that is discussed in your coordination with dieticians and practice assistants?

- ◯ Yes
- ◯ No

Q28 Are you familiar with the fact that as of 1 January 2019 you can refer patients to combined lifestyle interventions (GLI’s)?

◯ Yes

◯ No

Q29 Do you refer to combined lifestyle interventions (GLI’s)?

◯ Yes

◯ No

Q30 If I **don't refer** patients to combined lifestyle interventions (GLI’s) / or **less than I would like**, this is because of...

	Totally disagree	Disagree	Neutral	Agree	Totally agree
Lack of knowledge about GLI’s	◯	◯	◯	◯	◯
Lack of trust in GLI’s	◯	◯	◯	◯	◯
Lack of time	◯	◯	◯	◯	◯
Lack of motivation	◯	◯	◯	◯	◯
Lack of GLI’s nearby	◯	◯	◯	◯	◯
Insecurity about financial compensation in the future	◯	◯	◯	◯	◯
Preference for referring to familiar professionals within or	◯	◯	◯	◯	◯

outside my
practice

Otherwise, namely
____

Q31 When you give your patients lifestyle advice, how often do you provide follow-up support to them, for example, follow-up appointment, follow-up call, and/or medication reduction?

- ○ Never
- ○ Barely
- ○ Sometimes
- ○ Often
- ○ Always

Q32 When your patients undergo a lifestyle intervention, how often do you taper off medication?

- ○ Never
- ○ Barely
- ○ Sometimes
- ○ Often
- ○ Always

Q33 How do you rate your own lifestyle?

- ○ 1
- ○ 2
- ○ 3
- ○ 4

○ 5

○ 6

○ 7

○ 8

○ 9

○ 10

General questions (The questionnaire now takes less than half a minute)

Q34 What is your gender?

○ Female

○ Male

Q35 What is your age?

____

Q36 How many working days do you work on average as a general practitioner?

____

Q37 What is the postcode of your general practice? (only the numbers)

____

Q38 Are you working in a...

○ Solo practice

○ Solo practice in health centre

○ Independent duo practice

○ Duo practice in health centre

○ Independent group practice

○ Group practice in health centre

Q39 Are you familiar with Vereniging Arts & Leefstijl?

- No I do not know it
- Yes I know it, but no member and have not been to a training
- Yes I know it, member, but not have been to a training
- Yes I know it, member and have been to a training

This is the end of the questionnaire. If you want to win the gift voucher of 25 euros, you can leave your email address below.
